# Bioactive Lipids in Health and Disease

**DOI:** 10.3390/biom10121698

**Published:** 2020-12-21

**Authors:** Monica Bari, Tiziana Bisogno, Natalia Battista

**Affiliations:** 1 Department of Experimental Medicine, University of Rome Tor Vergata, 00133 Rome, Italy; 2 Endocannabinoid Research Group, Institute of Translational Pharmacology, National Research Council, 00133 Rome, Italy; 3 Faculty of Bioscience and Technology for Food, Agriculture and Environment, University of Teramo, 64100 Teramo, Italy

Although the primordial concept of lipids is associated with the role they play as key components of the cell membrane, growing research in the field of bioactive lipids and lipidomic technologies proves the prominent role of these molecules in other biological functions. Nowadays, over 100,000 bioactive lipids, including many different classes of this family (i.e., sphingolipids, fatty acids, and sterols), have been identified as signaling molecules in the regulation of complex pathways and molecular mechanisms implicated in both physiologic homeostasis and disease pathology, such as arthritis, cancer, heart disease, obesity, and neurodegenerative disorders. Therefore, a deeper comprehension of the existing link between bioactive lipids and cellular functions, from cell signaling to intercellular communication, and metabolic and gene regulation, is required to likely unveil the role of these lipids as diagnostic and/or prognostic biomarkers of different diseases.

This Special Issue includes four original research articles and five reviews that focus on the role of bioactive lipids and/or enzymes directly involved in their metabolism as new targets for future therapeutic intervention in different pathologies.

The paper of Thomas et al. [1] explores the role of the enzyme long-chain acyl-CoA synthetase 1 (ACSL1) in tumor necrosis factor-α (TNF-α)-mediated production of granulocyte-macrophage colony-stimulating factor (GM-CSF) by tumor cells. Overexpression of GM-CSF is involved in the pathogenesis of inflammatory diseases and is associated with tumor growth and progression. Using a series of modern biochemical methods, the authors show that ACSL1 is strongly required for TNF-induction of GM-CSF by breast cancer metastatic cells and acts upstream of MAPK and NF-κB signaling pathways, pointing to ACSL1 as a potential novel therapeutic target for cancer therapy.

Nokhala et al. [2] investigate the α-glucosidase inhibitory potential of the hydromethanolic extracts obtained from the leaves of *Tetracera scandens*, a Southeast Asian herb traditionally used for the cure of diabetes mellitus. Through a metabolomics approach, the authors identify the metabolites, belonging to different chemical classes, that elicit this activity. The data are also corroborated by an in silico docking study that predicts the binding affinities and the possible interactions of the ligand–enzyme complexes.

The study of Semaev et al. [3] examines the association of CETP polymorphism gene RS708272 with lipid changes and risk of myocardial infarction in a cohort of Western Siberian individuals. Although previous studies have extensively investigated the CETP gene polymorphism in various white populations, this paper unveils interesting gender associations that should be taken into consideration in the development of primary-prevention programs for cardiovascular diseases and, thus, needs further investigation.

The novel role played by finasteride, the prototypical inhibitor of steroid 5α-reductase, on emotional behaviors related to stress reactivity and mood regulation in rodents is highlighted in the original research article of Godar et al. [4]. Evidence in humans suggests that finasteride exerts serious emotional adverse effects with symptoms including depression, feelings of anxiety, and social phobia. To date, the mechanism underlying these aspects has not been elucidated, and this study offers interesting findings that should be crucial to understand whether and how finasteride negatively affects brain function in order to reduce impulsive behaviors.

In the context of gangliosides, Cavdarli et al. [5] present the most recent progress on the specific functions that these lipids have in normal tissues and malignant tumors. In particular, they summarize the characteristic expression of gangliosides in neuro-ectoderm-derived cancers, as well as other cancers, and report the dual opposite roles played by gangliosides modified with sialic acid residues in cancer cells, opening new, intriguing perspectives on the clinical practice as potential therapeutic targets for cancer.

The review by Mikhailova et al. [6] offers a comprehensive and up-to-date overview of the current state-of-the art of the genetic basis of familial hypercholesterolemia. The authors provide a description of gene structure and protein function as well as a presentation of the evidence for a role of the gene/protein for the familial hypercholesterolemia phenotype. For the general internist, endocrinologist, cardiologist, clinical geneticist, and so forth, without being a profound expert, this article offers a readable review for efficient and effective continuing medical education.

In their review, Gill et al. [7] describe the Advanced Glycation End products (AGEs) as a striking link between modern diet and health. Glycation of proteins is a post-translational modification that forms temporary adducts, which, following crosslinking and rearrangement, generate AGEs. Therefore, understanding post-translational modifications and their derivatives is likely the key to unlocking the mechanisms and physiology of various metabolic syndromes.

The review by Marrone and Coccurello [8] deals with an original topic on the role of dietary fatty acids and the microbiota-brain communications in neuropsychiatric diseases, taking into account the mechanisms by which lipids may modify gut microbiota. Establishing a possible link of dietary fatty acids to a modified microbiome is an important approach to improving the identification of microbiota- and neuropsychiatric diseases-associated biomarkers to potentiate both early diagnosis and personalized medicine. It would also provide a valuable contribution to the scientific community.

Battista et al. [9] conduct a very interesting and comprehensive review of the mechanisms for the biosynthesis and inactivation of the *N*-acyl amino acids, as well as of their molecular targets, with a particular focus on the role played by *N*-acyl-Glyines and *N*-Acyl-Serines in biological processes. These mediators, belonging to the complex lipid signaling system now known as endocannabinoidome, could have a crucial effect in terms of their therapeutic potential.

## References

[1] Thomas R., Al-Rashed F., Akhter N., Al-Mulla F., Ahmad R. (2019). ACSL1 Regulates TNFα-Induced GM-CSF Production by Breast Cancer MDA-MB-231 Cells. Biomolecules.

[2] Nokhala A., Siddiqui M.J., Ahmed Q.U., Ahamad Bustamam M.S., Zakaria Z.A. (2020). Investigation of α-Glucosidase Inhibitory Metabolites from *Tetracera scandens* Leaves by GC– MS Metabolite Profiling and Docking Studies. Biomolecules.

[3] Semaev S., Shakhtshneider E., Orlov P., Ivanoshchuk D., Malyutina S., Gafarov V., Ragino Y., Voevoda M. (2019). Association of RS708272 (*CETP* Gene Variant) with Lipid Profile Parameters and the Risk of Myocardial Infarction in the White Population of Western Siberia. Biomolecules.

[4] Godar S.C., Cadeddu R., Floris G., Mosher L.J., Mi Z., Jarmolowicz D.P., Scheggi S., Walf A.A., Koonce C.J., Frye C.A. (2019). The Steroidogenesis Inhibitor Finasteride Reduces the Response to Both Stressful and Rewarding Stimuli. Biomolecules.

[5] Cavdarli S., Groux-Degroote S., Delannoy P. (2019). Gangliosides: The Double-Edge Sword of Neuro-Ectodermal Derived Tumors. Biomolecules.

[6] Mikhailova S., Ivanoshchuk D., Timoshchenko O., Shakhtshneider E. (2019). Genes Potentially Associated with Familial Hypercholesterolemia. Biomolecules.

[7] Gill V., Kumar V., Singh K., Kumar A., Kim J.-J. (2019). Advanced Glycation End Products (AGEs) May Be a Striking Link Between Modern Diet and Health. Biomolecules.

[8] Marrone M.C., Coccurello R. (2020). Dietary Fatty Acids and Microbiota-Brain Communication in Neuropsychiatric Diseases. Biomolecules.

[9] Battista N., Bari M., Bisogno T. (2019). *N*-Acyl Amino Acids: Metabolism, Molecular Targets, and Role in Biological Processes. Biomolecules.

